# Diagnostic Support in Dentistry Through Artificial Intelligence: A Systematic Review

**DOI:** 10.3390/bioengineering12111244

**Published:** 2025-11-13

**Authors:** Alessio Danilo Inchingolo, Grazia Marinelli, Arianna Fiore, Liviana Balestriere, Claudio Carone, Francesco Inchingolo, Massimo Corsalini, Daniela Di Venere, Andrea Palermo, Angelo Michele Inchingolo, Gianna Dipalma

**Affiliations:** 1 Department of Interdisciplinary Medicine, University of Bari “Aldo Moro”, 70121 Bari, Italy; alessiodanilo.inchingolo@uniba.it (A.D.I.); grazia.marinelli@uniba.it (G.M.); arianna.fiore@uniba.it (A.F.); liviana.balestriere@uniba.it (L.B.); claudio.carone@uniba.it (C.C.); massimo.corsalini@uniba.it (M.C.); daniela.divenere@uniba.it (D.D.V.); giannadipalma@tiscali.it (G.D.); 2 Department of Experiment Medicine, University of Salento, 73100 Lecce, Italy; andrea.palermo@unisalento.it; 3 Department of Biomedical, Surgical and Dental Sciences, Milano University, 20122 Milan, Italy

**Keywords:** artificial intelligence, oral health, diagnosis, dentistry, clinical decision-making, radiography

## Abstract

**Background/Objectives**: The integration of artificial intelligence (AI) into dental diagnostics is rapidly evolving, offering opportunities to improve diagnostic precision, reproducibility, and accessibility of care. This systematic review examined the clinical performance of AI-based diagnostic tools in dentistry compared with traditional methods, with particular attention to radiographic assessment, orthodontic classification, periodontal disease detection, and other relevant specialties. **Methods**: Comprehensive searches of PubMed, Scopus, and Web of Science were carried out for articles published from January 2015 to June 2025, in accordance with PRISMA guidelines. Only English-language clinical studies investigating AI applications in dental diagnostics were included. Fifteen studies fulfilled the inclusion criteria and underwent quality appraisal and risk-of-bias assessment. **Results**: Across diverse dental fields, AI systems showed encouraging diagnostic capabilities. Radiographic algorithms enhanced lesion detection and anatomical landmark identification, while machine learning models successfully classified malocclusions and periodontal status. Photographic image analysis demonstrated potential in geriatric and preventive care. However, methodological variability, limited sample sizes, and the absence of external validation constrained generalizability. Study quality ranged from high to moderate, with some reports affected by bias or incomplete data reporting. **Conclusions**: AI holds considerable promise as an adjunct in dental diagnostics, particularly for imaging-based evaluation and clinical decision support. Broader clinical adoption will require methodological harmonization, rigorous multicenter trials, and validation of AI systems across diverse patient populations.

## 1. Introduction

Artificial intelligence (AI) is increasingly being recognized as a practical tool in dentistry, with applications extending from image-based diagnostics to risk prediction models [1]. Rather than remaining a distant or purely theoretical concept, AI has already been implemented in specific diagnostic workflows, supported by a growing body of research that demonstrates its utility in processing complex datasets, detecting subtle radiographic features, and synthesizing large volumes of clinical information [2,3,4,5,6,7]. These capabilities offer opportunities to improve diagnostic precision, reproducibility, and efficiency across a range of dental specialties [8,9].

Recent investigations have explored AI applications in key diagnostic challenges, ranging from the detection of periapical lesions on radiographs to the classification of skeletal malocclusions and the prediction of periodontal disease risk [10,11]. In several diagnostic domains—particularly those involving repetitive tasks or prone to inter-operator variability—AI performance has equaled or surpassed that of experienced clinicians [12]. Beyond improving diagnostic accuracy, AI holds potential to expand access to care, providing decision-support tools for practitioners in remote or resource-limited settings and enabling earlier screening and monitoring pathways [13,14,15,16].

One of AI’s most compelling attributes is its adaptability [17,18,19]. Whether applied to anatomical landmark detection in cone beam computed tomography or to photographic analysis of the oral cavity in geriatric patients, AI models can be tailored to diverse clinical contexts [20,21,22]. Nonetheless, current development is hindered by notable limitations: many systems are trained on small, single-institution datasets, rarely undergo external validation, and often rely on retrospective designs with heterogeneous labeling protocols [23,24,25,26]. These factors, combined with the absence of standardized methodological frameworks, can limit clinical applicability [27,28,29,30,31]. Additionally, as AI tools begin to exert greater influence on diagnostic decision-making, concerns regarding transparency, bias, and ethical deployment must be addressed [32,33,34,35].

Despite these constraints, the trajectory of AI integration into dental diagnostics is unmistakable [36,37,38,39,40]. It is poised to become a routine component of diagnostic workflows, complementing rather than replacing human clinical judgment. When implemented thoughtfully, AI can strengthen decision-making, reduce diagnostic variability, and contribute to equitable and high-quality patient care.

**Figure 1:**
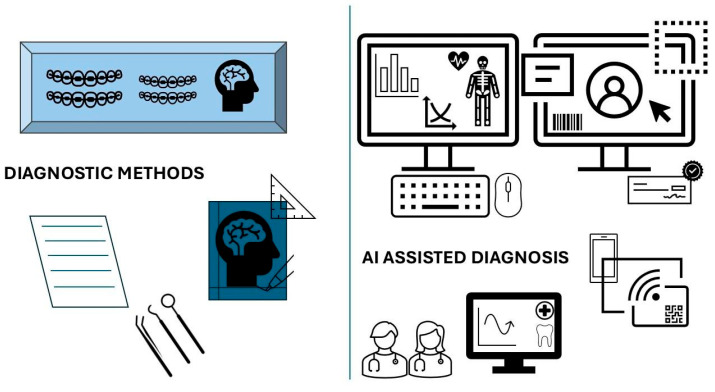
Schematic illustration of diagnostic methods with AI assistance.

This review synthesizes recent evidence on AI applications in dental diagnostics, with emphasis on radiographic interpretation, orthodontic classification, periodontal and peri-implant disease screening, and geriatric oral health. By highlighting both the current capabilities and the existing shortcomings of these technologies, we aim to provide a balanced, evidence-based perspective to guide their integration into everyday practice. In doing so, we also seek to outline realistic expectations for the role of AI in dentistry—where it currently stands and where it may be headed (Figure 1).

## 2. Materials and Methods

### 2.1. PICO Question

The PICO approach is typically used to evaluate the effect of an intervention on a specific condition, in this case, the use of AI and its implication in dentistry.

*In patients undergoing dental diagnostic procedures (P), does the application of artificial intelligence technologies—such as machine learning and deep learning (I)—enhance diagnostic accuracy and efficiency (C) compared to conventional diagnostic methods performed by dental professionals (O)?*

By comparing AI-based approaches with traditional diagnostic techniques, this review seeks to evaluate the effectiveness and clinical value of AI in dental diagnostics, particularly in terms of performance, reliability, and its potential to support clinical decision-making.

### 2.2. Protocol and Registration

Our search was performed following the method of Preferred Reporting Items for Systematic Reviews and Meta-Analysis (PRISMA) guidelines and registered in the International Prospective Register of Systematic Review Registry guidelines (PROSPERO ID: CRD420251104023).

### 2.3. Search Processing

The electronic databases PubMed, Scopus and Web of Science were searched to find papers that matched our topic dating from 1 January 2015, up to 30 June 2025. The Medical Subject Headings (MESH) terms entered in search engines were: (“artificial intelligence” OR “machine learning” OR “deep learning” OR “neural networks”) AND (“dental” OR “dentistry” OR “oral health” OR “odontology”) AND (“diagnosis” OR “diagnostic” OR “detection” OR “screening”) (Table 1).

### 2.4. Inclusion and Exclusion Criteria

The inclusion criteria were the following: (1) English language; (2) any type of observational study, i.e., retrospective cohort, prospective cohort, cross-sectional and randomized controlled trials; (3) open access; (4) articles concerning the use of AI in dental diagnostics.

The exclusion criteria were the following: (1) other languages except English; (2) reviews, meta-analysis and case–control; (3) off-topic articles; (4) in vivo studies; (5) in vitro studies.

### 2.5. Data Processing

The reviewers (A.F., L.B., C.C.) screened the records according to the inclusion and exclusion criteria. Doubts have been resolved by consulting the senior reviewer (F.I.). The selected articles were downloaded into Zotero.

## 3. Results

### 3.1. Study Selection and Characteristics

A total of 8532 records were identified using the keywords (“artificial intelligence” OR “machine learning” OR “deep learning” OR “neural networks”) AND (“dental” OR “dentistry” OR “oral health” OR “odontology”) AND (“diagnosis” OR “diagnostic” OR “detection” OR “screening”). When applicable, the automatic filters entered were only in English, only clinical studies and free full text. The consulted databases were PubMed (2616), Scopus (3864) and Web of Science (2052).

**Figure 2:**
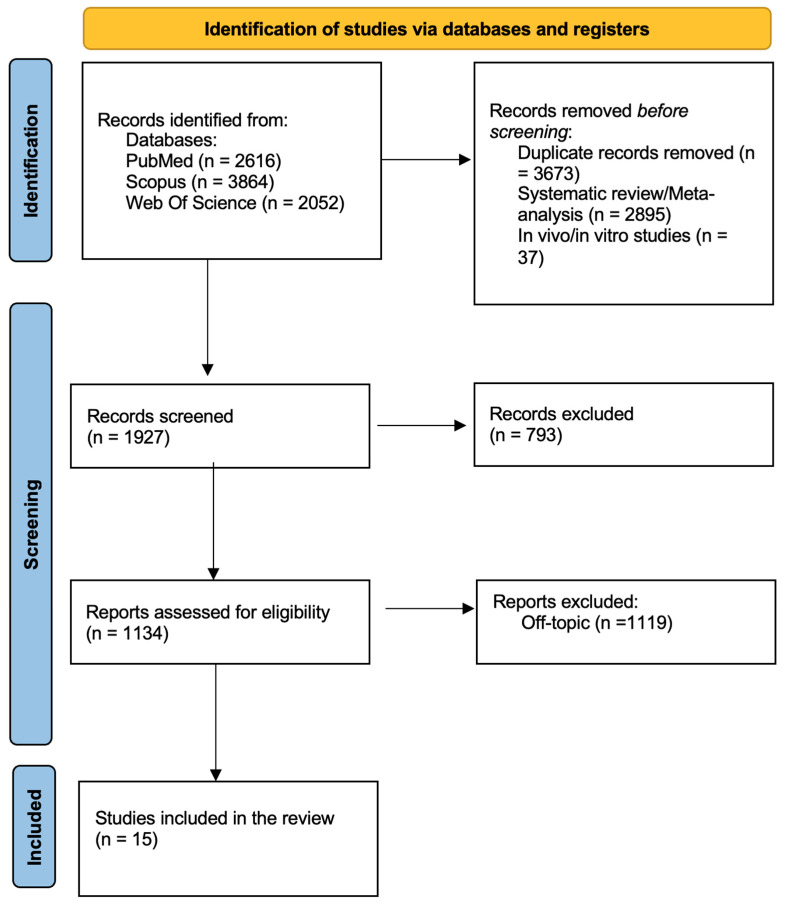
PRISMA flow diagram illustrating the study selection process.

After screening, 3673 duplicated articles, 2895 systematic reviews/meta-analysis and 37 in vivo/in vitro studies were excluded. Then 793 articles were excluded by the analysis of titles, leading to 1134 records assessed for eligibility. After eligibility assessment, 15 studies (Table 2) were included in the final analysis, and 1119 were excluded by the abstract. The process is summarized in Figure 2.

### 3.2. Quality Assessment and Risk of Bias of Included Articles

A structured risk of bias evaluation was performed across seven domains to evaluate the methodological quality of the included studies: (D1) bias resulting from confounding; (D2) bias resulting from exposure measurement; (D3) bias in the selection of study participants; (D4) bias resulting from post-exposure interventions; (D5) bias resulting from missing data; (D6) bias resulting from outcome measurement; and (D7) bias in the selection of the reported result. The approach was adapted from the ROBINS-I tool to better fit the characteristics of diagnostic AI studies, where exposures correspond to algorithmic applications rather than clinical interventions. The seven domains were retained, but the terminology and examples were adjusted to reflect diagnostic accuracy outcomes and dataset-related biases [56].

Every study was assessed and categorized by domain as either low risk, high risk, extremely high risk, some concerns, or no information. Most studies (60%) had some issues in at least one area, especially with regard to participant selection (D3), outcome assessment (D6), and confounding factors (D1). This was particularly true for cross-sectional and observational research, where blinding was frequently not disclosed and participant allocation was not randomized.

The five studies (Navarro-Fraile et al., Wang et al., Al-Sarem et al., Pul et al., and Zhou et al.) were noteworthy for their robust methodologies, which included randomization, objective outcome measurement (e.g., CBCT, segmentation accuracy), and complete reporting. These studies were deemed to have an overall low risk of bias. 

On the other hand, three studies (Uribe et al., Muramatsu et al., and Pépin et al.) were judged to have a high overall risk of bias, mainly because of subjective outcome assessments, inadequate reporting, and poorly controlled confounding variables. Despite being systematic, the Uribe et al. study had high risk in several categories since it lacked objective measures for evaluating exposure and outcomes as well as uniform criteria for dataset inclusion.

Some issues were found with the remaining studies (e.g., Stillhart, Deng, Ghensi, Yıldız), especially with regard to the use of subjective pain or diagnostic scores without uniform calibration across examiners, potential selection bias, and the transparency of data management. 

Overall, even though a few of studies showed excellent methodological rigor, the sample’s variation in design quality and reporting procedures highlights the need for more uniformity in AI-focused diagnostic research in dentistry. When evaluating generalizability to clinical practice or interpreting pooled findings, risk of bias should be taken into account.

A summary of the risk of bias for each study is provided in Table 3.

## 4. Discussion

The integration of artificial intelligence (AI) into dental diagnostics represents a rapidly evolving frontier in clinical practice and research [57,58]. Across various branches of dentistry, AI has demonstrated the ability to improve diagnostic accuracy, reduce variability, and support clinical decision-making through automated data analysis [59,60]. This discussion synthesizes the findings from recent studies on AI applications in different dental specialties, highlighting progress, common challenges, and areas for future development.

### 4.1. Radiographic and Imaging Diagnostics

A significant portion of the included studies focused on the use of artificial intelligence in radiographic interpretation and anatomical landmark detection [42,50,61]. Pul et al. (2024) and Zhou et al. (2025) showed that AI support significantly improved the diagnostic accuracy of dentists in identifying periapical radiolucency, especially among less experienced clinicians [51,55,62]. Picoli et al. (2023) further demonstrated AI’s utility by comparing 3D AI-generated models with CBCT and panoramic imaging for third molar risk assessment, concluding that AI offers a reliable alternative for presurgical planning [52]. Wang et al. (2025) and Al-Sarem et al. (2024) explored AI’s role in CBCT analysis, particularly in automating mandibular landmark detection and dental structure segmentation, helping to simplify implant planning and asymmetry analysis [45,46]. These studies highlight how AI tools can support more precise and consistent image interpretation, regardless of clinical experience [63,64,65].

### 4.2. Orthodontics and Skeletal Malocclusion Assessment

AI applications in orthodontics were investigated in several studies [66,67,68]. Midlej et al. (2024) developed machine learning models with high accuracy (up to 0.99) for classifying skeletal class II and III malocclusions using cephalometric data [53]. Schwab et al. (2024), although not using AI, provided normative morphological data on the sella turcica in the Austrian population, useful as a reference for future AI model training [44,69]. Navarro-Fraile et al. (2024) applied AI-based segmentation tools to assess orthodontic treatment-related root resorption, showing comparable results to manual analysis [42,70,71]. These findings confirm the growing role of AI in orthodontic diagnosis and treatment planning [72,73,74].

### 4.3. Periodontology and Implantology

Deng et al. (2023) proposed an innovative strategy for periodontal screening by combining artificial intelligence with salivary biomarkers and self-reported indicators. Their findings demonstrated encouraging potential for non-clinical and rapid screening approaches, although diagnostic accuracy remained limited by the binary nature of traditional classification methods. In contrast, Ghensi et al. (2025) investigated the peri-implant microbiome through shotgun metagenomic analysis, revealing that a patient’s history of periodontitis plays a crucial role in shaping early biofilm formation around implants. Shotgun genomics (or shotgun metagenomics) is a DNA sequencing technique that analyzes all the genetic material present in a sample. The DNA is fragmented, sequenced, and reconstructed using computational algorithms, allowing the identification of all microorganisms present and the study of their diversity, providing a more comprehensive view compared to traditional targeted methods [47,48].

This study underscores the relevance of microbiome-based profiling for identifying early signs of peri-implant disease and tailoring preventive strategies. Taken together, these contributions illustrate how advanced computational tools and omics-based analyses are reshaping oral diagnostics. Artificial intelligence, when integrated with biological and microbial data, holds promise for more precise, personalized, and predictive models of screening and disease prevention in periodontal and peri-implant care.

### 4.4. Geriatric and Preventive Dentistry

Muramatsu et al. (2021) specifically addressed the needs of the aging population by developing convolutional neural networks (CNNs) capable of assessing oral health status from simple photographic images. Their system demonstrated strong performance in identifying markers such as poor oral hygiene, mucosal dryness, and other signs associated with progressive oral health deterioration. Importantly, this approach illustrates how AI-based image analysis can reduce reliance on resource-intensive clinical examinations, offering a viable solution for populations with limited access to dental professionals [49,75,76]. For elderly patients—who are often homebound, institutionalized, or living in remote areas—such tools could provide timely monitoring, early detection of oral health decline, and prompt referral for appropriate care. The study therefore highlights the broader potential of artificial intelligence in promoting equitable oral healthcare delivery, while also suggesting avenues for integrating CNN-based screening into telemedicine and community care settings [77,78,79].

### 4.5. Orofacial Pain and Temporomandibular Disorders

Yıldız et al. (2023) developed AI-based predictive models for diagnosing temporomandibular disorders (TMD) using clinical and psychological data [50,80,81]. Their ensemble model (Bagging MARS) achieved the best results, particularly in settings without radio-diagnostic tools. Stillhart et al. (2024) explored the use of automated facial recognition to detect dental pain expressions but found limited sensitivity to expression changes [41,49,51]. These studies open new possibilities for non-invasive diagnostic support in orofacial pain.

### 4.6. Sleep Medicine in Dentistry

Pépin et al. (2024) introduced an AI-based approach to analyse mandibular movements in patients with obstructive sleep apnoea (OSA) treated with oral appliances [54]. The model was able to distinguish sleep stages and respiratory events based on mandibular signals, suggesting a potential non-invasive, home-based alternative to traditional polysomnography [82,83,84,85,86,87,88].

### 4.7. Limitations and Future Directions

Despite the promising results, the studies analysed present several limitations [89,90,91,92,93,94,95]. Many had small sample sizes, such as those reported by Picoli, R. S., et al. (2021) and Pul, M., et al. (2022), limiting the generalizability of the findings. Some investigations, including Stillhart, A., et al. (2024), reported insufficient sensitivity of AI models to specific clinical cues like pain expressions. Several studies were based on retrospective data, for example, Muramatsu, C., et al. (2021) and Navarro-Fraile, J., et al. (2023), which may introduce bias and weaken causal inference. Furthermore, limited dataset standardization, as highlighted by Uribe, S., et al. (2024), and the lack of external validation represent critical obstacles to clinical translation [41,42,43,49,51,52]. Future research should prioritize multicentre trials, standardized data collection, and rigorous prospective validation [43,96,97]. Future research should prioritize multicenter trials, standardized data collection, and rigorous prospective validation [46]. Moreover, it is important to provide a broader interpretation of the results in the context of existing literature, highlighting how our findings fit within the current body of research on the role of artificial intelligence in dentistry. In this perspective, the integration between AI-based algorithms and computer software should also be explored, as exemplified by the *Co-Mask R-CNN* collaborative learning-based method for tooth instance segmentation, as well as the use of smartphone applications, which are emerging as valuable tools to support daily clinical practice [98,99]. These integrations represent potential future developments to enhance accessibility, efficiency, and personalization in dental care. Addressing model transparency and ethical considerations is also essential to foster trust and adoption in everyday dental practice.

### 4.8. Ethical, Medico-Legal, and Cognitive Considerations

Beyond the technical and methodological limitations, the integration of AI into dental practice also raises important medico-legal and ethical challenges. Issues related to data privacy and security are particularly relevant, as the use of sensitive patient data for training and deploying AI models requires strict compliance with regulations such as General Data Protection Regulation and Health Insurance Portability and Accountability Act, alongside robust anonymization and cybersecurity measures to protect against misuse [30,43]. Moreover, medico-legal accountability remains a critical question: in cases of diagnostic errors or treatment failures, it is unclear whether liability should rest with the clinician, the AI developer, or the healthcare institution. Another important concern is the potential for automation bias, where clinicians may over-rely on AI-generated outputs and neglect their own clinical judgment. Such overdependence could lead to errors, especially when AI models encounter atypical or underrepresented cases not adequately captured in training datasets [100,101]. To mitigate these risks, AI systems should be implemented as decision-support tools rather than autonomous diagnostic agents, with clear guidelines promoting clinician oversight, transparency, and interpretability. This critical reflection emphasizes that the future of AI in dentistry depends not only on technological advances but also on establishing ethical frameworks, medico-legal safeguards, and education strategies to support responsible and balanced adoption [75,102,103].

## 5. Conclusions

The application of artificial intelligence in dental diagnostics holds considerable potential for improving diagnostic accuracy, efficiency, and clinical decision-making. Recent evidence highlights that AI-based tools, particularly those employed for radiographic analysis and automated classification of oral conditions, can help reduce inter-operator variability and enhance diagnostic reliability. These advantages are especially relevant in settings with limited resources or among less experienced clinicians, where AI can act as a valuable decision-support tool.

Nevertheless, the current body of research is limited by heterogeneous methodological quality, the absence of standardized datasets, and insufficient external validation, which constrain the generalizability and clinical implementation of these findings. Future research should prioritize multicentre, prospective studies with harmonized protocols, rigorous external validation, and transparent AI algorithms. Addressing these challenges will be essential to ensure the safe, effective, and ethically responsible integration of AI into routine dental practice, ultimately supporting improved patient outcomes and more equitable oral healthcare delivery.

## Figures and Tables

**Table 1:** Article screening strategy.

KEYWORDS	A: Artificial Intelligence; Machine learning; Deep learning; Neural networks B: Dental; Dentistry; Oral health; Odontology; C: Diagnosis; Diagnostic; Detection; Screening
BOOLEAN INDICATORS	“A” AND “B” AND “C”
TIMESPAN	From 1 January 2015, to 30 June 2025
ELECTRONIC DATABASES	PubMed, Scopus and Web of Science

**Table 2:** Analysis of the studies included in the discussion section.

Authors	Study Type	Study Sample	Aim of Study	Materials and Methods	Conclusions
Stillhart et al. (2024) [41]	Observational study	57 patients	Evaluate Automated Face Coding software’s effectiveness in detecting facial expressions related to dental pain	Facial expressions recorded and analyzed at baseline and post-treatment using AFC software. Pain assessed with VAS.	AFC software showed limited sensitivity to changes in pain-related expressions; more research needed for integration in diagnostics.
Navarro-Fraile et al. (2024) [42]	Randomized clinical trial	43 patients	Assess root resorption using AI-aided segmentation with different orthodontic forces	CBCT images segmented manually and with AI; resorption compared in control and experimental groups	AI showed comparable accuracy to manual in length detection but less sensitivity in volume loss; promising for clinical use.
Uribe et al. (2024) [43]	Observational study	131,028 records screened; 16 datasets analyzed	Evaluate publicly available dental image datasets for AI development.	Systematic search across databases; analysis of dataset characteristics and FAIR metrics.	Limited publicly available datasets and inconsistent metadata; better quality and access needed for robust AI in dentistry.
Schwab et al. (2024) [44]	Observational study	208 cephalometric radiographs	Assess Sella Turcica morphology and correlation with skeletal class	Cephalometric analysis with demographic correlation using statistical methods	Identified normative ST values for Austrian population; useful for orthodontic diagnostics and future AI integration.
Wang et al. (2025) [45]	Experimental study	400 CBCT scans (360 training, 40 validations, 50 test)	Automate mandibular landmark detection using AI for midsagittal plane construction	Deep learning models (PointRend, PoseNet) used to segment mandible and identify landmarks	Accurate automatic landmark detection and segmentation support use in mandibular asymmetry analysis.
Al-Sarem et al. (2024) [46]	Experimental study	500 CBCT images	Enhance tooth region detection using pretrained deep learning models	Six pretrained CNNs applied to segmented CBCT data; models tested with and without segmentation	DenseNet169 achieved best performance; supports automated implant planning systems.
Deng et al. (2023) [47]	Cross-sectional diagnostic study	408 participants	Develop machine learning tool to screen periodontal health using non-clinical parameters	Random forest models using questionnaire, biomarkers, and demographic data	High accuracy in classifying periodontal stages; promising for population screening applications.
Ghensi et al. (2025) [48]	Observational study	102 individuals, 158 samples	Evaluate plaque microbiome as a biomarker for peri-implant diseases using shotgun metagenomics	Shotgun sequencing of submucosal plaque; machine learning for taxonomic/functional profile analysis	Identified disease-specific microbial signatures; supports future diagnostic and personalized treatment strategies.
Muramatsu et al. (2021) [49]	Retrospective observational study	3201 images from 114 older patients	Construct CNN models to assess oral status of elderly using image data	CNNs trained to classify oral health features into assessment scores	Models demonstrated high diagnostic accuracy for multiple oral conditions in elderly; enhances remote assessment capability.
Yıldız et al. (2023) [50]	Cross-sectional observational study	228 participants (125 TMD, 103 non-TMD)	Predict TMD using machine learning based on clinical features	20+ ML models trained on physical and psychological metrics; best model identified by validation	Bagging MARS model achieved best performance; useful for preliminary diagnosis in clinics lacking imaging.
Pul et al. (2024) [51]	Randomized controlled trial	30 dentists, 50 panoramic radiographs	To evaluate the impact of AI on diagnostic accuracy and confidence for periapical radiolucency	Cross-over design with AI-aided and unaided assessments; CBCT as reference standard	AI reduced false positives and improved diagnostic accuracy and confidence, especially for junior dentists
Picoli et al. (2023) [52]	Within-patient controlled trial	25 patients with bilateral M3M removal	To assess risk of inferior alveolar nerve injury using 3D AI-driven models compared to CBCT and PANO	3D models created from CBCT using AI platform; examiners scored IAN risk from different modalities	3D AI had similar sensitivity to CBCT; promising tool for pre-surgical planning of M3M removal
Midlej et al. (2024) [53]	Observational study	502 patients (Class II and III malocclusion)	To establish ML models for classifying skeletal malocclusions in Arab orthodontic patients	Cephalometric data analyzed with PCA and ML models including LDA, SVM, KNN, RF, CART	High accuracy (up to 0.99) achieved in classifying skeletal classes using ML models with cephalometric inputs
Pépin et al. (2024) [54]	Observational study	Obstructive sleep apnea patients (exact N not specified)	Automate mandibular jaw movement analysis to monitor oral appliance treatment in OSA patients	Machine learning applied to mandibular jaw movement signals to classify sleep/OSA events	Automated MJA analysis provided reliable classification of respiratory events and sleep stages
Zhou et al. (2025) [55]	Experimental study	50 panoramic radiographs, evaluated by 30 dentists	Evaluate AI’s impact on dentist performance in identifying periapical radiolucency	Dentists interpreted images with/without AI assistance; diagnostic metrics compared	AI improved diagnostic accuracy and reduced inter-observer variability, particularly for less experienced dentists

**Table 3:** Risk of bias of the articles.

Authors	D1	D2	D3	D4	D5	D6	D7	Overall
Stillhart et al. (2024) [41]	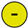	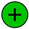	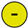	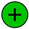	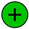	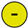	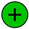	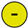
Navarro-Fraile et al. (2024) [42]	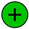	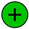	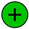	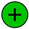	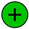	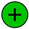	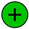	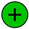
Uribe et al. (2024) [43]	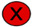	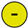	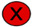	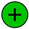	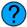	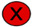	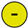	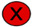
Schwab et al. (2024) [44]	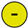	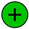	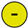	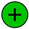	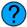	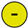	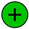	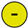
Wang et al. (2025) [45]	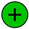	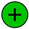	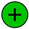	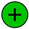	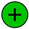	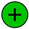	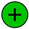	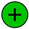
Al-Sarem et al. (2024) [46]	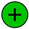	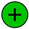	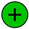	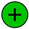	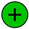	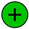	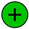	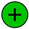
Deng et al. (2023) [47]	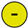	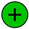	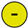	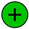	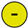	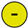	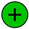	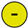
Ghensi et al. (2025) [48]	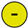	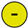	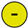	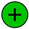	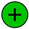	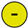	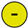	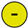
Muramatsu et al. (2021) [49]	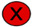	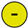	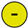	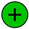	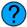	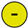	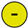	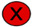
Yıldız et al. (2023) [50]	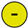	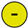	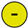	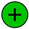	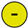	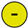	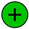	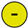
Pul et al. (2024) [51]	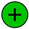	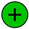	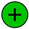	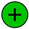	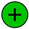	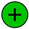	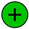	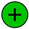
Picoli et al. (2023) [52]	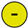	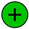	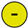	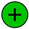	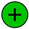	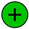	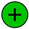	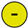
Midlej et al. (2024) [53]	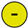	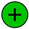	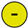	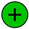	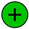	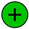	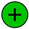	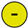
Pépin et al. (2024) [54]	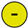	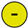	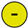	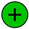	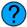	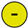	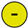	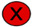
Zhou et al. (2025) [55]	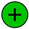	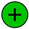	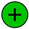	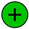	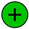	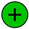	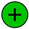	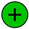

Domains: D1: Bias due to confounding. D2: Bias arising from measurement of the exposure. D3: Bias in selection of participants into the study (or into the analysis). D4: Bias due to post-exposure interventions. D5: Bias due to missing data. D6: Bias arising from measurement of the outcome. D7: Bias in Selection of the Reported Result. 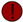 Very High; 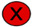 High; 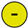 Some Concerns; 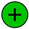 Low; 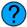 No information.

## Data Availability

Data sharing is not applicable.

## Abbreviations

The following abbreviations are used in this manuscript:

AFC	Automated Face Coding
AI	Artificial Intelligence
CBCT	Cone Beam Computed Tomography
CNN	Convolutional Neural Network
IAN	Inferior Alveolar Nerve
M3M	Mandibular Third Molar
MESH	Medical Subject Headings
MJA	Mandibular Jaw Movement Analysis
ML	Machine Learning
OSA	Obstructive Sleep Apnea
PCA	Principal Component Analysis
RF	Random Forest
SVM	Support Vector Machine
TMD	Temporomandibular Disorders
VAS	Visual Analogue Scale
